# Proteasome Dysfunction and Aggregation-Prone Proteins in Neurodegenerative Diseases: From Mechanisms to Therapeutic Opportunities

**DOI:** 10.3390/ijms27135730

**Published:** 2026-06-25

**Authors:** Youngwon Kim, Yong-Keun Jung

**Affiliations:** School of Biological Sciences, Seoul National University, 1 Gwanak-ro, Gwanak-gu, Seoul 08826, Republic of Korea

**Keywords:** proteasome, neurodegenerative diseases, proteostasis, protein aggregation

## Abstract

Neurodegenerative diseases are characterized by the accumulation of misfolded and aggregation-prone proteins, reflecting a failure of proteostasis. The ubiquitin–proteasome system (UPS), a major pathway for selective intracellular protein degradation, is essential for maintaining neuronal protein homeostasis. Proteasome dysfunction has been implicated in several major neurodegenerative disorders, including Alzheimer’s disease (AD), Parkinson’s disease (PD), amyotrophic lateral sclerosis (ALS), and Huntington’s disease (HD), although its extent and mechanisms vary across disease contexts. In this review, we examine current evidence for proteasome dysfunction in neurodegeneration and discuss how disease-associated proteins impair proteasome function through direct inhibition, defective substrate processing, and sequestration into protein aggregates. We also address the contribution of oxidative stress, neuroinflammation, and aging to proteasome dysregulation. Finally, we highlight emerging therapeutic strategies aimed at restoring proteasome function, including pharmacological activation, modulation of proteasome assembly and stability, and targeted protein degradation approaches. Understanding the context-dependent nature of proteasome dysfunction will be important for developing effective proteostasis-based therapies.

## 1. Introduction

Neurodegenerative diseases comprise a heterogeneous group of disorders that progressively affect distinct neuronal populations [1]. Although Alzheimer’s disease (AD), Parkinson’s disease (PD), amyotrophic lateral sclerosis (ALS), and Huntington’s disease (HD) differ in their clinical and neuropathological features, many of them share a disruption of proteostasis. Accumulation of misfolded or aggregation-prone proteins places a sustained burden on intracellular protein quality control systems, making protein degradation pathways particularly relevant to disease progression [1,2]. One of the main intracellular systems responsible for protein quality control is the ubiquitin–proteasome system (UPS). In this pathway, proteins selected for degradation are typically tagged with ubiquitin and then delivered to the 26S proteasome for unfolding and proteolysis. This process is especially important in neurons, which must maintain proteostasis over long periods of time despite limited regenerative capacity [1,2].

Observations from both human tissue and experimental models have repeatedly linked neurodegeneration with ubiquitin-positive inclusions and altered proteasome function [3,4,5,6,7,8,9]. What those findings mean mechanistically, however, is still not straightforward. In some settings, proteasome activity is clearly reduced. In others, the changes are more subtle, region-restricted, or dependent on disease stage and experimental model [6,10,11]. The underlying causes are also likely to be mixed rather than singular. Misfolded proteins may act directly on the proteasome, but broader cellular disturbances, such as oxidative stress and neuroinflammation, are also likely to shape proteasome function in disease [12,13,14]. Against this background, the remaining sections examine how proteasome dysfunction is implicated in neurodegenerative disease, summarize the mechanisms that have been proposed, and discuss current therapeutic strategies targeting the proteasome system.

## 2. Proteasome Dysfunction in Neurodegenerative Diseases

### 2.1. Evidence for Proteasome Impairment in Neurodegeneration

Proteasome dysfunction has been examined in neurodegenerative disease for many years, and studies have reported reduced proteasome activity in the affected nervous tissues. Even so, the impaired pattern is not identical across disorders. The degree of change, the regions involved, and the mechanisms proposed differ depending on the disease being studied. Evidence for the reduced proteasome activity is well documented in Alzheimer’s disease (AD), particularly in the hippocampus and cortex [3,15]. Related findings have also been described in Parkinson’s disease (PD), most notably in the substantia nigra [4]. In amyotrophic lateral sclerosis (ALS), reduced activity has been detected in the spinal cord, especially in regions enriched for motor neurons [5]. On the other hand, Huntington’s disease (HD) is less straightforward. Although some analyses of HD patient-derived brain tissues have shown lower proteasome activity than in non-HD patients [6], results from experimental systems have been more mixed. In cell and animal models, proteasome function is often preserved or only modestly altered, and expanded huntingtin fragments can still be processed by the proteasome under some experimental conditions [10,11].

Changes in proteasome composition have also been described. Several studies have reported reduced expression of proteasome subunits in neurodegenerative conditions. In AD, transcriptomic analyses have shown progressive downregulation of genes encoding proteasome subunits as the disease advances [16]. Similar changes have also been reported at the protein level in human dementia brain tissue. For example, reduced expression of the 19S ATPase subunit Rpt6 (PSMC5) has been observed in the cortical tissue from patients with neurodegenerative dementia, including AD [17]. In PD, selective loss of 20S proteasome α-subunits has been detected in dopaminergic neurons of the substantia nigra [18]. In ALS, reduced levels of the catalytic β5 (PSMB5) subunit have been found in the spinal cord [5]. Taken together, the available studies indicate that proteasome dysfunction is a recurring feature of neurodegenerative disease, although it does not appear in exactly the same form in every disorder. It is still uncertain whether impaired proteasome function acts as an early pathogenic event or develops later as a consequence of protein aggregation, oxidative stress, and broader cellular damage. This distinction remains important for understanding how proteasome dysfunction should be interpreted in the context of disease progression.

### 2.2. Proteasome Dysfunction and Protein Aggregation

A defining pathological feature of many neurodegenerative diseases is the accumulation of ubiquitin-positive protein aggregates. The frequent detection of proteasome components within these inclusions has long suggested a link between protein aggregation and impaired proteasomal protein clearance. However, the mechanistic relationship between these phenomena remains complex and not fully resolved. In AD, pathologic hallmark neurofibrillary tangles and senile plaques have been shown to contain ubiquitinated proteins along with proteasome components, indicating that elements of the proteasome machinery are associated with sites of pathological protein deposition [7]. Proteostasis defects have also been implicated in frontotemporal dementia (FTD), including both tau- and TAR DNA-binding protein 43 (TDP-43)-associated subtypes, in which pathological protein aggregates are closely linked to proteasome dysfunction and impaired protein clearance [19,20,21]. Similarly, in PD, Lewy bodies—the characteristic intracellular inclusions—contain multiple components related to protein degradation pathways, including proteasome-associated factors, such as PA700 (19S regulatory particle) and PA28 (proteasome activator), as well as ubiquitin pathway enzymes [7,22]. In ALS, motor neurons frequently exhibit ubiquitin-positive cytoplasmic inclusions, including skein-like and Lewy body-like structures. These inclusions are also immunoreactive for p62, a ubiquitin-binding adaptor protein that is involved in protein quality control and degradation pathways [8]. Ubiquitinated protein deposits are frequently observed in prion disease pathology [23]. Experimental studies further demonstrated that disease-associated prion protein (PrP) aggregates directly impair proteasome function, resulting in defective protein clearance and inhibition of 26S proteasome activity [24]. Consistent with these findings, prion-infected experimental models exhibit reduced proteasome activity and early UPS dysfunction [25]. In HD, mutant huntingtin (HTT) accumulates in neuronal intranuclear inclusions and dystrophic neurites, which are strongly immunoreactive for ubiquitin [9].

The detection of proteasome components within these protein aggregates does not by itself establish proteasome failure. Instead, these findings may reflect impaired proteasomal degradation, active recruitment of proteasomes to the aggregation-prone substrates, or sequestration of proteasome complexes within the inclusions. This pattern highly supports a reciprocal relationship between protein aggregation and proteasome dysfunction. In general, the reduced proteasomal clearance may favor the accumulation of protein aggregates, whereas the aggregated proteins may further impair proteasome function through direct inhibition of the proteasome, inefficient processing of substrate proteins, or physical sequestration of proteasomes.

In addition, proteasome dysfunction has also been linked to genetic factors that regulate ubiquitin-dependent protein quality control pathways. Mutations in ubiquitin shuttle or processing proteins, such as ubiquilin-2 (UBQLN2), impair the delivery of ubiquitinated substrates to the proteasome and disrupt efficient protein degradation, thereby contributing to neurodegenerative disease pathogenesis [26]. In addition, genetic alterations affecting proteasome-associated regulatory components, including haploinsufficiency of Rpn5 (PSMD12), further demonstrate that disruption of proteasome-related pathways can directly impact neuronal function and lead to neurological disease phenotypes [27]. Although these genetic defects do not primarily target proteasome catalytic core activity in typical late-onset neurodegenerative diseases, they highlight that the impairment of proteasome-linked protein quality control can contribute to neurodegeneration through convergent proteostasis failure mechanisms.

## 3. Mechanisms of Proteasome Impairment by Disease-Associated Proteins

### 3.1. Inhibition of Proteasome Activity via Direct Interaction

Direct inhibition of the proteasome by disease-associated protein species has been reported in several neurodegenerative disease contexts. In AD, oligomeric amyloid-β (Aβ) has been found to inhibit proteasome activity through direct interaction with the proteasome complex [12]. Available data suggest that oligomeric Aβ interferes with conformational changes required for proteasome function, with possible effects on gate opening and substrate translocation. Aβ has also been proposed to act as a proteasome substrate under certain experimental conditions, which could potentially influence degradation capacity [28]. In addition, the aggregated tau species, including oligomeric and fibrillar forms, have been reported to associate with the proteasome and impair proteasome-mediated degradation [19]. Pathological tau disrupts 26S proteasome function in experimental systems. A comparable effect has been described for α-synuclein in PD. Both oligomeric and fibrillar α-synuclein species bind to proteasome subunits and suppress proteolytic activity [29,30]. However, the precise molecular basis remains unresolved.

Depending on the experimental setting, proteasome inhibition may involve impaired substrate recognition, altered translocation, or other disruption of proteasome function. As with Aβ and tau, the inhibitory effect of α-synuclein appears to depend in part on its conformational state. Overall, these studies support direct interaction between misfolded protein species and the proteasome as one mechanism of proteasome impairment. Even so, it is still difficult to determine how much this mechanism contributes to proteasome dysfunction in vivo, particularly in human disease tissue. Importantly, direct inhibition and defective substrate processing are not entirely distinct mechanisms. In many cases, impaired substrate processing may itself lead to functional inhibition of the proteasome, particularly when degradation intermediates accumulate or stall within the proteasome.

### 3.2. Proteasome Impairment via Substrate-Dependent Processing Defects

Impaired substrate processing is mechanistically linked to proteasome dysfunction, as inefficient or stalled degradation can compromise overall proteasome activity. Although impaired substrate processing is a common consequence of proteasome dysfunction, the underlying mechanisms vary depending on the biochemical properties of each pathogenic protein. Tau is an intrinsically disordered protein that can be degraded by the proteasome. Pathological modifications, however, can reduce the efficiency of this process. In particular, hyperphosphorylated tau, a major pathological species in AD, has been reported to impair proteasomal degradation of tau [31]. Phosphorylated tau species show reduced susceptibility to proteasomal degradation, which may prolong their persistence within cells and favor the accumulation of tau species that are more difficult to clear. Such inefficient processing may further challenge cellular protein quality-control systems and contribute to proteostasis imbalance [32]. α-Synuclein is also degraded by the proteasome under physiological conditions [33]. Under pathological conditions, however, α-synuclein appears to be processed less efficiently. In PD, aggregation-prone α-synuclein species, including familial mutant forms such as A30P and A53T, impair the degradation of proteasome reporter substrates in cellular models [34]. These findings suggest that pathogenic α-synuclein interferes with proteasome-mediated substrate turnover and compromises the degradation of multiple UPS substrates.

Mutant forms of superoxide dismutase 1 (SOD1), which are linked to familial ALS, are likewise subject to proteasomal degradation under physiological conditions [35]. Mutant SOD1 expression, however, has been shown to disrupt proteasome-mediated protein degradation. In cellular models, aggregation-prone SOD1 variants impair the degradation of proteasome reporter substrates [36]. Transgenic mice expressing SOD1G93A accumulate UPS reporter proteins in spinal motor neurons [37], suggesting that misfolded SOD1 imposes a chronic burden on the UPS and may overwhelm proteasomal degradation capacity through substrate overload. HTT is also subject to proteasomal degradation under physiological conditions [38]. Expansion of the polyglutamine (polyQ) tract in mutant HTT associated with HD, however, markedly alters its proteasomal processing [39]. PolyQ-expanded HTT fragments, which are prone to aggregation in HD, are processed inefficiently by the proteasome and can become kinetically stalled during degradation [39]. The intrinsic properties of expanded polyQ sequences limit efficient proteasomal cleavage and may interfere with substrate processing, thereby contributing directly to proteasome stasis [39,40].

### 3.3. Sequestration of Proteasomes into Protein Aggregates

Protein aggregates may impair proteasome function not only through direct inhibition or defective substrate processing, but also by physically trapping proteasome complexes. One recent study addressed this mechanism using aggregates formed by the 25 kDa C-terminal fragment of TDP-43, referred to as TDP-25 [22]. In neurons and cultured cells expressing aggregation-prone TDP-25, cytoplasmic inclusions showed gel-like biophysical properties and were strongly enriched in 26S proteasomes [22]. Cryo-electron tomography showed marked accumulation of proteasomes within these inclusions, whereas other large macromolecular complexes, such as ribosomes, were largely excluded [22]. Structural analysis further indicated that proteasomes trapped in TDP-25 inclusions predominantly adopt substrate-processing conformations, suggesting that they become stalled during the degradation cycle [22]. Functional assays were consistent with these structural observations. Expression of TDP-25 led to the accumulation of the proteasome substrates and disruption of cellular proteostasis [22]. These findings support a model in which protein aggregates can sequester proteasomes and interfere with their function. How broadly this mechanism operates across human neurodegenerative disease, however, is still not clear.

**Figure 1:**
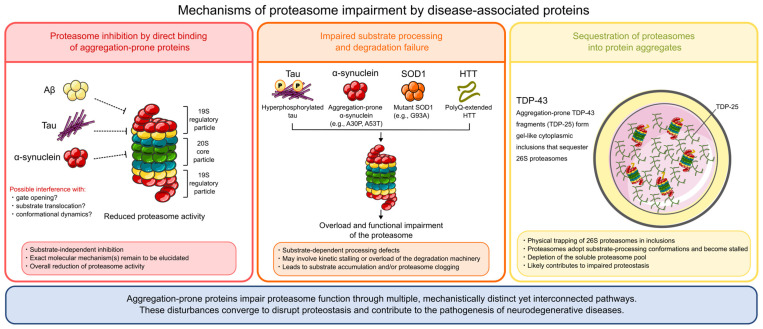
Aggregation-prone proteins disrupt proteasome function through multiple mechanisms, including direct inhibition of proteasomal activity, impairment of substrate processing, and sequestration of proteasome components. These defects collectively reduce degradation efficiency and lead to proteostasis disruption in neurodegenerative diseases. This figure was created using icons from Bioicons.com. Elements were either used under the CC0 license or adapted/modified from icons provided by Servier Medical Art under a CC BY 3.0 license. Abbreviations: amyloid-β (Aβ), superoxide dismutase 1 (SOD1), huntingtin (HTT), TAR DNA-binding protein 43 (TDP-43), and the 25 kDa C-terminal fragment of TDP-43 (TDP-25). Dashed lines indicate inhibitory effects, and arrows indicate the direction of the associated processes. Colors are used solely for visual clarity.

In addition, other regulatory mechanisms employing the post-translational modifications could largely influence proteasome function under various pathophysiological conditions. In particular, the phosphorylation of proteasome subunits has been shown to regulate proteasome activity in multiple experimental systems [41,42]. However, direct evidence showing the linkage of the disease-associated phosphorylation events to proteasome inhibition in neurodegenerative disorders remains limited and needs to be thoroughly explored. Further studies are required to determine how pathological signaling pathways affect proteasome function in vivo. The mechanisms by which aggregation-prone proteins impair proteasome function are summarized in Figure 1.

## 4. Cellular Dysregulation of the Proteasome in Neurodegeneration

### 4.1. Altered Proteasome Function by Oxidative Stress

Oxidative stress is a common feature of many neurodegenerative diseases and can strongly affect proteasome function. Reactive oxygen species (ROS) generated under oxidative conditions modify cellular proteins, leading to the accumulation of oxidatively damaged or misfolded species that require efficient degradation to preserve proteostasis [13,43]. The proteasome is central to this process, as oxidatively modified proteins are preferentially degraded by the 20S proteasome in an ATP- and ubiquitin-independent manner [44]. Oxidative stress not only affects substrate proteins but also acts directly on the proteasome itself. Proteasome subunits are susceptible to oxidative modification, which can reduce catalytic activity and compromise structural integrity. The 26S proteasome appears to be particularly vulnerable under oxidative conditions and can undergo structural destabilization [45]. Oxidative stress can affect proteasome function in a stress intensity- and duration-dependent manner. Under mild or transient oxidative stress, proteasome function may be preserved as part of adaptive cellular stress responses [13]. During this stage, oxidatively damaged proteins are preferentially degraded by the 20S proteasome in an ATP- and ubiquitin-independent manner, thereby supporting cellular proteostasis [13,44,45]. In response to cellular stress, the proteasome system may undergo adaptive remodeling, including reversible dissociation of the 26S proteasome into the 20S core particle and the 19S regulatory particle [13,46]. This process increases the pool of free 20S proteasomes, which are more resistant to oxidative damage and can efficiently degrade oxidized and partially unfolded proteins without ATP-dependent substrate unfolding [44,45]. However, under prolonged or severe oxidative stress, adaptive mechanisms become insufficient. Excessive ROS promotes the accumulation of heavily oxidized, cross-linked, and aggregation-prone proteins that are degraded inefficiently and tend to accumulate within cells [13,44]. Simultaneously, proteasome subunits themselves become targets of oxidative modification. Oxidative damage to proteasome components impairs proteasome assembly, destabilizes the 26S complex, and reduces catalytic activity [45,47]. As a result, both substrate burden and direct proteasome damage contribute to progressive proteostasis failure. Evidence consistent with these mechanisms has been reported in disease contexts, including impaired degradation of oxidatively modified proteins in AD and reduced proteasome activity in PD [48,49].

Collectively, these findings support a hierarchical model in which oxidative stress initially induces adaptive modulation of proteasome function, but ultimately promotes proteasome dysfunction when oxidative damage becomes chronic or excessive.

### 4.2. A Relationship Between Proteasome Function and Neuroinflammation

Neuroinflammation is a common pathological feature of many neurodegenerative diseases. In the central nervous system, inflammatory responses are driven mainly by activated microglia and astrocytes, which release pro-inflammatory cytokines, chemokines, and reactive mediators that can influence neuronal survival and proteostasis [14]. The proteasome system, particularly the immunoproteasome, is increasingly recognized as an important regulator of inflammatory responses in the central nervous system [50]. Immunoproteasomes are highly expressed in activated glial cells and are induced under inflammatory conditions to regulate inflammatory signaling pathways [50,51,52]. Proteasome-dependent degradation of the regulatory proteins in intracellular signaling pathways controls activation of transcription factors, such as nuclear factor kappa B (NF-κB). Degradation of the NF-κB inhibitor IκB is a critical step in this process, as it permits NF-κB nuclear translocation and subsequent transcription of pro-inflammatory genes [53]. Proteasome activity thus influences microglial activation and cytokine production. In this context, the immunoproteasome catalytic subunit β5i (PSMB8) regulates neuroinflammation in microglia, and its inhibition reduces the levels of inflammatory mediators, including TNF-α and inducible nitric oxide synthase [52].

Inflammatory signaling can also reshape proteasome composition and function. Pro-inflammatory cytokines, particularly interferon-γ, induce expression of the immunoproteasome catalytic subunits β1i (PSMB9), β2i (PSMB10), and β5i (PSMB8), which are incorporated into newly assembled proteasomes under inflammatory conditions [51]. Compared with the standard proteasome, immunoproteasomes have altered catalytic properties and substrate preferences, which may support the degradation of oxidized or damaged proteins during inflammatory stress. Experimental studies in microglia and other cell types have shown that inflammatory stimulation promotes immunoproteasome assembly and changes overall proteasome composition [51]. Links between proteasome function and neuroinflammatory responses have been described in several neurodegenerative disorders. In AD, reactive glial cells surrounding Aβ plaques show increased immunoproteasome expression and activity, and immunoproteasome inhibition reduces inflammatory marker expression in microglia [54,55]. In PD, accumulation of misfolded α-synuclein activates microglia and stimulates inflammatory signaling pathways [56,57]. In ALS, TDP-43 aggregation has been linked to immune activation [58]. In HD models, mutant HTT has also been linked to glial activation and inflammatory responses in the brain [59].

**Figure 2:**
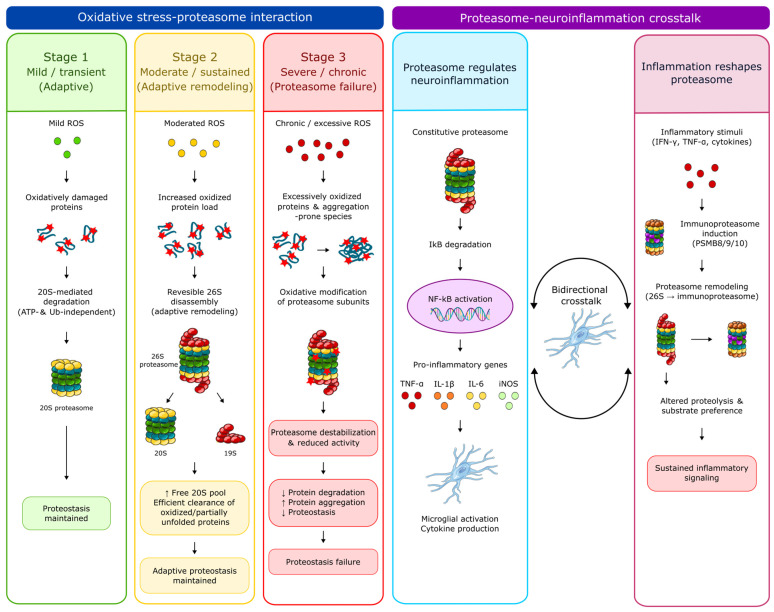
Oxidative stress and neuroinflammation modulate proteasome function through interconnected mechanisms. Mild oxidative stress promotes adaptive proteasome responses, whereas prolonged oxidative stress impairs proteasome function and proteostasis; inflammatory signaling and immunoproteasome induction establish a bidirectional relationship between proteasome function and neuroinflammation. This figure was created using icons from Bioicons.com. Elements were either used under the CC0 license or adapted/modified from icons provided by Servier Medical Art (CC BY 3.0), Database Center for Life Science (DBCLS; CC BY 4.0), and SMART (Servier Medical Art; CC BY 4.0). All modifications were made by the authors. Abbreviations: reactive oxygen species (ROS), adenosine triphosphate (ATP), ubiquitin (Ub), nuclear factor kappa B (NF-κB), inhibitor of κB (IκB), tumor necrosis factor alpha (TNF-α), interleukin-1 beta (IL-1β), interleukin-6 (IL-6), inducible nitric oxide synthase (iNOS), interferon gamma (IFN-γ), proteasome subunit beta type-8 (PSMB8), proteasome subunit beta type-9 (PSMB9), and proteasome subunit beta type-10 (PSMB10). Stars indicate oxidized species. Upward and downward arrows indicate increases and decreases, respectively. Colors are used solely for visual clarity.

These observations support a bidirectional relationship between proteasome function and neuroinflammation. The proteasome regulates inflammatory signaling through controlled degradation of key signaling proteins, while inflammatory stimuli alter proteasome composition through induction of the immunoproteasome. If this balance is not properly maintained, it may contribute to both proteostasis impairment and sustained neuroinflammation during neurodegeneration. The interplay between oxidative stress, proteasome dysfunction, and neuroinflammation is summarized in Figure 2.

### 4.3. Decline of Proteasome During Aging

Aging is the major risk factor for most neurodegenerative diseases and is closely associated with a gradual decline in proteostasis capacity. The incidence of major neurodegenerative disorders increases markedly with age. The incidence of major neurodegenerative disorders increases markedly with age. AD and PD show a strong age-dependent increase in incidence, supporting the idea that aging-associated cellular changes are central to disease susceptibility [60,61,62]. At the molecular level, aging is accompanied by progressive deterioration of the proteostasis network, including protein synthesis, folding, and degradation pathways, among which the proteasome plays a key role in maintaining protein quality control [2]. Evidence from multiple studies indicates that proteasome activity declines with age across different tissues, including the central nervous system. Early biochemical studies reported a marked reduction in proteasome activity in several tissues of aged rodents, suggesting that proteasome impairment is a general feature of aging [63,64]. Similar changes have also been observed in the nervous system. In the spinal cord of aged rats, both proteasome activity and proteasome expression were significantly reduced compared with young animals [65]. More recent studies have further shown that proteasome activity declines in the aging brain and is associated with impaired proteasome assembly and accumulation of ubiquitinated proteins [66].

Aging is also accompanied by an increased burden of misfolded, oxidized, and aggregation-prone proteins, which places additional stress on the proteasome system and reduces its effective degradation capacity [67]. Among age-associated factors, oxidative stress appears to be one of the major contributors to proteasome dysfunction. Proteasome subunits are susceptible to oxidative modifications that impair catalytic activity and structural integrity, while oxidatively damaged and cross-linked proteins are degraded inefficiently by the proteasome [63,68]. Emerging evidence suggests that epigenetic alterations contribute to age-associated decline in proteostasis primarily through broad transcriptional reprogramming during aging. Age-related epigenomic remodeling, including changes in DNA methylation, histone modifications, and chromatin organization, has been shown to reshape gene expression programs involved in cellular stress responses and proteostasis maintenance [69]. Although direct epigenetic regulation of individual proteasome subunit genes in aging remains incompletely defined, proteasome function may be indirectly affected through stress-responsive transcriptional programs and broader proteostasis networks governed by chromatin-associated regulatory factors, including histone deacetylases (HDACs) and sirtuins, which are key modulators of aging-associated cellular homeostasis and protein quality control [69,70]. Recent work in aged animal models is also consistent with this view. Proteasome activity is reduced in the aging brain, including in regions such as the hippocampus, and enhancement of proteasome function has been reported to improve cognitive performance [71]. These findings support a functional link between proteasome activity and age-related neuronal decline. Aging-associated proteasome decline may also increase neuronal vulnerability to disease-associated proteotoxic stress. Neurons are long-lived cells and depend heavily on efficient proteasome function to maintain protein homeostasis. As proteasome activity declines with age, the ability to clear misfolded and damaged proteins becomes more limited. Under these conditions, additional stressors, such as the accumulation of aggregation-prone proteins, may exceed the already reduced proteasome capacity and promote protein aggregation and cellular dysfunction.

Overall, aging is associated with proteasome decline at multiple levels, including catalytic activity, subunit expression, and structural integrity. This decline likely lowers the capacity of neurons to maintain proteostasis and may increase susceptibility to neurodegenerative disease.

## 5. Therapeutic Targeting of the Proteasome

### 5.1. Pharmacological Activation of the Proteasome via Improving Substrate Accessibility

Because proteasome dysfunction is implicated in neurodegenerative diseases, pharmacological enhancement of proteasome activity has emerged as a potential strategy to promote the clearance of misfolded and aggregation-prone proteins [72,73]. Rather than altering proteasome abundance or assembly, most activation strategies aim to increase the catalytic efficiency or improve substrate accessibility of the existing proteasome complexes. One of the best-characterized approaches involves inhibition of the proteasome-associated deubiquitinating enzyme USP14 [74], which negatively regulates proteasome function by trimming ubiquitin chains and delaying substrate degradation. Pharmacological inhibition of USP14 with the small molecule IU1 enhances proteasome activity and accelerates the degradation of ubiquitinated substrates while also promoting clearance of aggregation-prone proteins, such as tau, in cellular models [74]. Similar effects have been reported with USP14-targeting aptamers, supporting its role as a regulatory node in proteasome activation [75].

Small molecules that directly stimulate proteasome activity have also been identified, particularly those targeting the 20S proteasome. These compounds generally enhance substrate access to the catalytic chamber and, in some cases, promote opening of the proteasome gate, thereby facilitating degradation of intrinsically disordered or partially unfolded proteins in a ubiquitin-independent manner [76,77]. Naturally occurring compounds have likewise been reported to modulate proteasome activity. Several polyphenolic molecules, including oleuropein and related dietary compounds, enhance proteasome-mediated degradation and improve cellular proteostasis, at least in part, through indirect mechanisms involving oxidative stress and stress-response pathways [78,79]. Despite these advances, several limitations remain. Much of the current evidence for pharmacological proteasome activation is derived from in vitro or cellular systems, and its therapeutic efficacy in vivo remains less well established [72]. Proteasome activation is not uniform across substrates, with intrinsically disordered proteins being more readily degraded. In addition, broad enhancement of proteasome activity may lead to unintended degradation of regulatory proteins and disruption of cellular homeostasis [73]. Future efforts will therefore need to focus on achieving more selective and context-dependent activation of proteasome function.

### 5.2. Regulation of Proteasome Assembly and Structural Integrity

Proteasome activity is influenced not only by catalytic function but also by assembly and structural organization. Proper formation of the 26S proteasome is required for efficient substrate processing [80,81,82,83]. Disruption of these processes can impair proteasome function even when the catalytic activity of individual subunits is preserved. Notably, phosphorylation is a major regulatory mechanism. Activation of cAMP-dependent protein kinase A (PKA) induces phosphorylation of the proteasome subunits, including Rpn6 (PSMD11), and is associated with increased stability of the 26S complex and enhanced proteasome activity [41,42,84]. This modification supports proteasome activity without necessarily increasing proteasome abundance. In neuronal models, activation of this pathway has been linked to reduced accumulation of tau and improved proteasome function [19].

Further, pharmacological activation of the cAMP–PKA pathway has been achieved using multiple classes of compounds that elevate intracellular cAMP levels [42]. Forskolin increases cAMP production and activates PKA [41,42]. Phosphodiesterase inhibitors, such as rolipram, prevent cAMP degradation and prolong signaling [19]. Membrane-permeable cAMP analogs, including dibutyryl-cAMP, directly stimulate this pathway in cellular systems [41]. In addition, proteasome composition can be altered. In contrast to the PKA-mediated stabilization, small molecule TCH-165 has been reported to promote disassembly of the 26S proteasome, leading to an increased pool of free 20S core particles and enhanced degradation of intrinsically disordered proteins [85,86]. This shift changes substrate processing rather than directly increasing catalytic activity. These observations indicate that proteasome function is regulated at multiple levels, including stability, assembly, and composition.

### 5.3. Targeted Protein Degradation as a Proteasome-Based Therapeutic Strategy

Targeted protein degradation (TPD) encompasses a group of therapeutic strategies designed to eliminate disease-associated proteins through endogenous cellular degradation pathways. Among these, UPS-dependent approaches have attracted particular interest as the proteasome-based strategies for selective protein clearance. Rather than inhibiting protein activity, these approaches promote ubiquitination of a target protein by recruiting an E3 ubiquitin ligase, which then directs the protein to the proteasome for degradation [87]. Because many degrader molecules can act catalytically, a single molecule may support repeated cycles of target engagement and degradation, making it possible to lower protein abundance efficiently in both cellular systems and in vivo models [88]. Among neurodegeneration-related targets, tau was one of the earliest proteins tested in this framework. Peptide-based proteolysis-targeting chimera (PROTAC)-like molecules were shown to induce ubiquitination and proteasomal degradation of endogenous tau in neuronal cell models, with a corresponding reduction in tau levels [87]. In the same experimental context, tau clearance was also associated with reduced cytotoxicity [87].

This concept has since been applied to other aggregation-prone proteins. In HD models, small-molecule degraders were reported to promote selective ubiquitination and degradation of polyglutamine-expanded huntingtin [89]. Pathological tau has also been targeted successfully in patient-derived neuronal models, where PROTAC-mediated ubiquitination reduced disease-associated tau species [90]. Similar work on α-synuclein has shown that small-molecule degraders can lower intracellular α-synuclein burden and mitigate aggregation-associated toxicity in cellular systems, although both proteasomal and lysosomal pathways may contribute depending on the degrader design [91]. At the same time, the success of proteasome-dependent degradation is influenced by the biochemical state of the target protein. Soluble or only moderately misfolded proteins appear to be more accessible to this strategy, whereas highly aggregated or fibrillar species are likely to be less efficiently processed by the proteasome [89,90,91]. This limitation is particularly relevant in neurodegenerative disease, where pathogenic proteins often accumulate in structurally heterogeneous forms. For that reason, autophagy-based degradation pathways are also being explored as complementary approaches, although a detailed discussion of those strategies is beyond the scope of this section [92].

On the other hand, there are practical barriers to therapeutic application in TPD. In particular, delivery across the blood–brain barrier, degrader selectivity, and the availability of E3 ligases suitable for neuronal tissues remain important translational challenges for proteasome-dependent targeted protein degradation approaches [93]. Taken together, these studies support TPD as a proteasome-based strategy for lowering pathogenic proteins, but they also make clear that its success in neurodegenerative disease will depend on improvements in molecular design, delivery, and target selectivity. Table 1 summarizes representative proteasome-based therapeutic strategies for neurodegenerative diseases.

## 6. Conclusions

Proteasome dysfunction is observed across many neurodegenerative diseases, but it does not appear in a uniform way. Its extent, direction, and mechanistic basis vary depending on disease context, brain region, and experimental system. A range of factors likely contribute to this dysregulation, including direct effects of misfolded proteins on the proteasome, impaired substrate processing, sequestration into aggregates, and broader cellular stresses, such as oxidative stress, neuroinflammation, and aging. An important unresolved issue is whether proteasome dysfunction is an initiating event in neurodegeneration or instead develops secondarily during disease progression. Clarifying this point will require better human-relevant model systems as well as improved methods for monitoring proteasome function in vivo. From a therapeutic standpoint, the most useful strategies will likely be those that modulate proteasome activity in a selective and context-dependent manner rather than simply enhancing proteolysis globally. Importantly, the optimal proteasome-targeted therapeutic strategy may differ among neurodegenerative diseases because the mechanisms underlying proteasome dysfunction are not identical. In AD, where impairment of 26S proteasome activity has been linked to pathological tau accumulation, approaches aimed at restoring proteasome function and promoting tau clearance may be particularly beneficial. In PD, therapeutic benefit may be achieved by combining proteasome-targeted interventions with approaches that reduce α-synuclein aggregation and oxidative or inflammatory stressors that further compromise proteasome activity. In ALS and TDP-43 proteinopathies, strategies that prevent proteasome sequestration and enhance the degradation of aggregation-prone proteins may be especially relevant. In HD, where mutant huntingtin can interfere with efficient proteasomal processing, interventions that improve substrate handling and facilitate mutant huntingtin clearance may represent promising therapeutic avenues. Therefore, future proteasome-based therapies will likely require disease-specific and mechanism-based approaches rather than a universal strategy applicable to all neurodegenerative disorders.

## Figures and Tables

**Table 1:** Representative proteasome-based therapeutic strategies for neurodegenerative diseases.

Strategy	Representative Examples	Mechanism	Development Stage
Enhancing substrate accessibility	IU1, USP14 aptamers, 20S activators, oleuropein	Enhanced proteasomal degradation through improved substrate accessibility	Cellular and preclinical studies
Regulation of proteasome assembly and stability	Forskolin, rolipram, dibutyryl-cAMP, PKA activation, TCH-165	Modulation of 26S stability or free 20S availability	Experimental studies
Targeted protein degradation	Tau-, huntingtin-, and α-synuclein-targeting degraders	E3 ligase-mediated proteasomal degradation	Preclinical proof-of-concept studies

## Data Availability

No new data were created or analyzed in this study. Data sharing is not applicable to this article.
